# From cultural competence to cross-cultural fluency: a framework for transnational medical education in an era of global health workforce mobility

**DOI:** 10.3389/fmed.2026.1809667

**Published:** 2026-05-14

**Authors:** Changiz Mohiyeddini

**Affiliations:** Department of Foundational Medical Studies, Oakland University William Beaumont School of Medicine, Oakland University, Rochester, MI, United States

**Keywords:** accreditation, cross-cultural fluency, cultural competence, faculty development, globalization, health equity, health workforce mobility

## Abstract

**Background:**

As medical education increasingly operates across national boundaries through institutional partnerships, offshore campuses, dual-degree programs, and digital learning platforms, the need for culturally responsive pedagogical frameworks has become urgent. Existing models of cross-cultural medical education were developed primarily for domestic settings and do not adequately address the complexities that arise when curricula, faculty, and learners simultaneously traverse multiple cultural, regulatory, and institutional contexts. This paper proposes a conceptual shift from cultural competence to cross-cultural fluency as a guiding principle for transnational medical education.

**Main body:**

Drawing on established frameworks in cross-cultural medical education, global health workforce literature, and transnational education governance, we present a three-tier framework—the Transnational Cross-Cultural Fluency (TCCF) model—designed to address the unique challenges of medical education delivered across borders. The first tier addresses organizational and structural barriers inherent in cross-border educational partnerships, including divergent accreditation standards, regulatory misalignment, and institutional power asymmetries. The second tier focuses on pedagogical and clinical challenges arising from the multiplication of cultural contexts when faculty from one national setting teach students from another who will practice in a third. The third tier examines individual-level processes of professional identity formation, cultural self-reflection, and adaptation when learners and educators navigate multiple institutional cultures simultaneously. For each tier, we propose specific strategies encompassing curriculum design, faculty development, assessment innovation, and governance mechanisms. We critically examine how digital platforms and technology-enhanced learning can serve as both enablers and complicators of cross-cultural fluency in transnational settings. We further address ethical dimensions including epistemic justice, the risk of cultural hegemony in cross-border partnerships, and the imperative for bidirectional knowledge exchange.

**Conclusion:**

The TCCF model extends existing cultural competence frameworks by explicitly accounting for the multi-layered cultural transactions that characterize transnational medical education. Moving beyond competence—which implies a finite endpoint—toward fluency—which denotes ongoing adaptive capacity—provides a more appropriate conceptual foundation for preparing healthcare professionals to deliver equitable care across a globalized healthcare landscape. Implementation of this framework requires coordinated effort across partnering institutions, accreditation bodies, and policymakers to ensure that cross-border medical education promotes rather than undermines health equity.

## Background

Healthcare systems worldwide now serve patient populations of extraordinary cultural, linguistic, and socioeconomic diversity (1–3). A substantial body of empirical evidence demonstrates that sociocultural factors, race, and ethnicity significantly influence health outcomes, treatment adherence, patient satisfaction, and clinical encounter quality (4–12), and that unacknowledged cultural differences between patients and providers can lead to misdiagnosis, medical errors, and suboptimal outcomes (9, 13–16).

In response to these challenges, the field of Cross-Cultural Medical Education (CCME) emerged to prepare physicians with the skills, knowledge, and attitudes necessary to navigate diverse patient populations (6, 17). Three key factors catalyzed this emergence: the imperative to serve increasingly diverse populations, the need to address persistent racial and ethnic disparities in healthcare, and accreditation standards established by bodies such as the Liaison Committee on Medical Education (LCME) requiring cross-cultural curricula (6, 18, 19). More recently, scholars have recognized that providers themselves bring their own cultural backgrounds, biases, and values into clinical encounters, necessitating bidirectional cultural awareness (6, 20, 21).

Importantly, the global health education community has not been silent on the challenges of cross-border educational partnerships. Frameworks such as the Fair Trade Learning Principles have articulated standards for reciprocity, mutual benefit, and community-driven partnerships in international educational exchanges (22). Similarly, the Monette Principles provide guidelines for equitable global health research and education collaborations, emphasizing transparent governance, shared decision-making, and accountability to local communities (23). These contributions have meaningfully advanced the field’s capacity to address partnership dynamics and power asymmetries. However, they were developed primarily to govern the ethics of bilateral partnerships and international exchanges rather than to address the specific pedagogical, accreditation, and identity-formation challenges that arise when medical education itself is delivered transnationally—where curricula, faculty, students, and regulatory frameworks simultaneously span multiple national systems.

However, these foundational frameworks were developed primarily for domestic educational settings in which faculty, students, and patients share a single national regulatory context. The landscape of medical education has since undergone a profound transformation. Medical schools now operate across national borders through diverse mechanisms: institutional partnerships and twinning arrangements, offshore branch campuses, dual-degree programs, clinical rotations conducted in international settings, and digital learning platforms that connect learners across continents (24–27). The World Federation for Medical Education (WFME) estimates that transnational medical education programs now operate in over 70 countries, enrolling hundreds of thousands of students in curricula that cross geographic, cultural, and regulatory boundaries (28, 29).

These transnational arrangements introduce cultural complexity of a fundamentally different order than what existing CCME frameworks were designed to address. In a domestic medical school, the primary cultural transaction occurs between a provider trained within one cultural system and a patient from another. In transnational medical education, the cultural dynamics multiply: faculty from one national and institutional culture teach students drawn from a second cultural context who may practice in a third, under accreditation frameworks that reflect a fourth set of cultural assumptions about what constitutes competent medical practice (30–32). This “triple cultural transaction”—and often a quadruple one when accreditation standards from the home institution’s country differ from those in the host country—exceeds the explanatory and prescriptive scope of existing models.

Furthermore, transnational medical education programs frequently operate within significant power asymmetries. Partnerships between institutions in high-income countries (HICs) and those in low- and middle-income countries (LMICs) can reproduce colonial dynamics in which knowledge flows unidirectionally, curricula are transplanted without adequate contextual adaptation, and local health priorities are subordinated to the partner institution’s pedagogical traditions (33–36). These dynamics have implications not only for educational quality but also for health equity, as graduates may be trained in frameworks that do not reflect the disease burden, healthcare infrastructure, or cultural health practices of the communities they will serve (37, 38).

Although this paper focuses on medical education as its primary context—given the specific accreditation structures, clinical training requirements, and workforce mobility patterns that characterize the medical profession—the TCCF framework is designed to be adaptable across health professions education. Transnational programs in nursing, allied health, pharmacy, public health, and interprofessional education face analogous cultural, structural, and pedagogical challenges when operating across national boundaries. The three-tier architecture of the TCCF model is profession-agnostic in its underlying logic: organizational power asymmetries, pedagogical adaptation needs, and individual identity formation processes arise whenever health professions education crosses national boundaries. The accreditation-specific elements of Tier 1 are most likely to require discipline-specific adaptation, while the pedagogical (Tier 2) and developmental (Tier 3) tiers are more directly transferable across professions. Empirical exploration of the framework’s applicability to these broader health professions education contexts represents a productive direction for future research.

This paper addresses this gap by proposing a conceptual framework—the Transnational Cross-Cultural Fluency (TCCF) model—that extends existing CCME approaches to explicitly account for the multi-layered cultural, regulatory, and institutional dynamics of cross-border medical education. We argue that the prevailing concept of cultural “competence,” which implies a discrete, achievable endpoint, is insufficient for the fluid, iterative, and contextually contingent demands of transnational practice. Instead, we propose “cross-cultural fluency” as a more appropriate organizing principle—one that denotes ongoing adaptive capacity, analogous to linguistic fluency, which enables practitioners to navigate novel cultural situations with sophistication rather than simply applying a fixed repertoire of learned cultural facts.

## From competence to fluency: a conceptual reorientation

The concept of cultural competence has served as the dominant paradigm in cross-cultural healthcare education for over two decades (6, 17, 39). Cultural competence frameworks typically emphasize the acquisition of specific knowledge about different cultural groups, the development of awareness regarding one’s own cultural biases, and the cultivation of skills for effective cross-cultural communication (20, 40). These frameworks have generated meaningful improvements in provider education and patient outcomes, and they remain valuable foundational resources (41, 42).

However, several scholars have identified limitations in the competence paradigm that become particularly acute in transnational contexts (43–45). First, cultural competence can inadvertently promote an essentialist view of culture in which patients and communities are understood through static group-level characteristics rather than as individuals embedded in dynamic, intersecting cultural systems (46). Second, the language of competence implies a threshold that can be definitively crossed—a state of having achieved sufficient cultural knowledge—which may foster complacency rather than ongoing learning (44). Third, and most critically for transnational education, competence frameworks were designed for relatively stable cultural dyads (provider–patient) rather than the multi-actor, multi-context cultural negotiations that characterize cross-border educational settings (47).

We propose “cross-cultural fluency” as an alternative conceptual anchor. Fluency, as a metaphor drawn from language acquisition, carries several advantages. It implies a capacity that develops continuously through immersive engagement rather than through discrete instructional episodes. It acknowledges that the same individual may exhibit different degrees of fluency across different cultural contexts. It foregrounds adaptive capacity—the ability to respond appropriately to novel cultural situations—over encyclopedic cultural knowledge. And it recognizes that true fluency emerges only through sustained, reciprocal engagement with cultural others, not through unidirectional instruction (48, 49).

This conceptual shift has practical implications for how transnational medical education programs are designed, delivered, and assessed. Rather than teaching students about specific cultural groups they may encounter, programs should cultivate the capacity to engage with unfamiliar cultural contexts through curiosity, humility, and structured reflection (50, 51). This orientation is particularly relevant in transnational settings, where the specific cultural contexts that graduates will encounter are inherently unpredictable and where the capacity for adaptive engagement is more valuable than any fixed body of cultural knowledge.

## Positioning TCCF within existing frameworks

The TCCF model builds on and complements existing contributions rather than seeking to replace them. Where the Fair Trade Learning Principles and Monette Principles provide essential ethical foundations for partnership governance (22, 23), the TCCF model operationalizes principles of equity and reciprocity within the specific institutional architecture of cross-border medical education. Where cultural humility foregrounds lifelong self-reflection and the redress of power imbalances in clinical encounters (44), the TCCF model extends this dispositional orientation to the organizational and structural level, addressing how institutions—not only individuals—can develop the conditions for genuine cross-cultural engagement. Where structural competency directs attention to the social and institutional determinants shaping health encounters (52), the TCCF model applies this structural lens to the educational enterprise itself, examining how accreditation systems, governance arrangements, and knowledge hierarchies shape the conditions under which cross-cultural fluency can develop. Where cultural safety, originating from the work of Māori nurse educator Dr. Irihapeti Ramsden in Aotearoa New Zealand, centers the analysis of power relationships between health professionals and those they serve and insists that safety be determined by the recipient of care rather than the provider (53, 54), the TCCF model extends this decolonizing orientation to the educational partnership itself, examining whose knowledge systems are privileged and whose are marginalized within transnational curricula. Where Leininger’s transcultural nursing theory of Culture Care Diversity and Universality provides a comprehensive framework for discovering and providing culturally congruent care through understanding both universal and diverse cultural care patterns (55, 56), the TCCF model moves beyond the clinical encounter to address the organizational, pedagogical, and identity-formation dimensions of educational systems that produce culturally fluent practitioners. And where global health competency frameworks delineate knowledge, skills, and attitudes for individual global health practice (57), the TCCF model situates individual development within the organizational and pedagogical systems that enable or constrain it. Table 1 provides a comparative overview of these frameworks and the specific contributions of the TCCF model.

**Table 1:** Comparative overview of cross-cultural frameworks and the TCCF model.

Framework	Core construct	Level of analysis	Primary context	Relationship to TCCF
Cultural humility (44)	Lifelong self-reflection; redress of power imbalances in clinical relationships	Individual (provider–patient dyad)	Domestic clinical encounters	TCCF incorporates humility as a dispositional foundation but extends to organizational and structural levels; adds multi-context navigation absent from dyadic models
Structural competency (52)	Recognition of institutional and social determinants shaping health encounters	Institutional/systemic	Domestic health systems	TCCF applies structural analysis to the educational enterprise itself—examining how accreditation, governance, and knowledge hierarchies shape cross-cultural learning conditions
Cultural safety (53, 54)	Decolonizing healthcare; analysis of power relationships; safety determined by care recipient; critical self-reflection on practitioner culture	Systemic/relational (provider–patient–system)	Indigenous health contexts; originated in Aotearoa New Zealand	TCCF extends the decolonizing and power-analysis orientation of cultural safety to transnational educational partnerships, examining whose knowledge systems are privileged in cross-border curricula and governance structures
Transcultural care/culture care diversity and universality (55, 106)	Culturally congruent care through understanding universal and diverse cultural care patterns; culture as embedded in care	Individual/community (nurse–patient–family)	Nursing practice across diverse cultural groups	TCCF moves beyond the clinical care encounter to address the organizational, pedagogical, and identity-formation dimensions of educational systems that produce culturally fluent practitioners; adds structural and governance tiers absent from care-focused models
Intercultural development continuum (104, 105)	Developmental progression from ethnocentrism to ethnorelativism	Individual cognitive/affective	General intercultural encounters	TCCF draws on developmental logic but adds organizational and pedagogical tiers for transnational education; situates individual development within institutional systems
Global health competency frameworks (57)	Knowledge, skills, attitudes for global health practice	Individual competency	Global health fieldwork/rotations	TCCF situates individual development within organizational and pedagogical systems; addresses sustained transnational education rather than episodic global health experiences
Fair trade learning/Monette principles (22, 23)	Partnership ethics; reciprocity; community accountability	Partnership governance	International exchanges and collaborations	TCCF operationalizes partnership ethics within the specific institutional architecture of cross-border medical education, adding pedagogical and developmental tiers
TCCF Model (present paper)	Cross-cultural fluency as ongoing adaptive capacity across organizational, pedagogical, and individual dimensions	Multi-level (organizational, pedagogical, individual)	Transnational medical education	Integrative framework synthesizing and extending elements from each of the above, calibrated for the multi-layered cultural transactions of cross-border medical education

**Figure 1:**
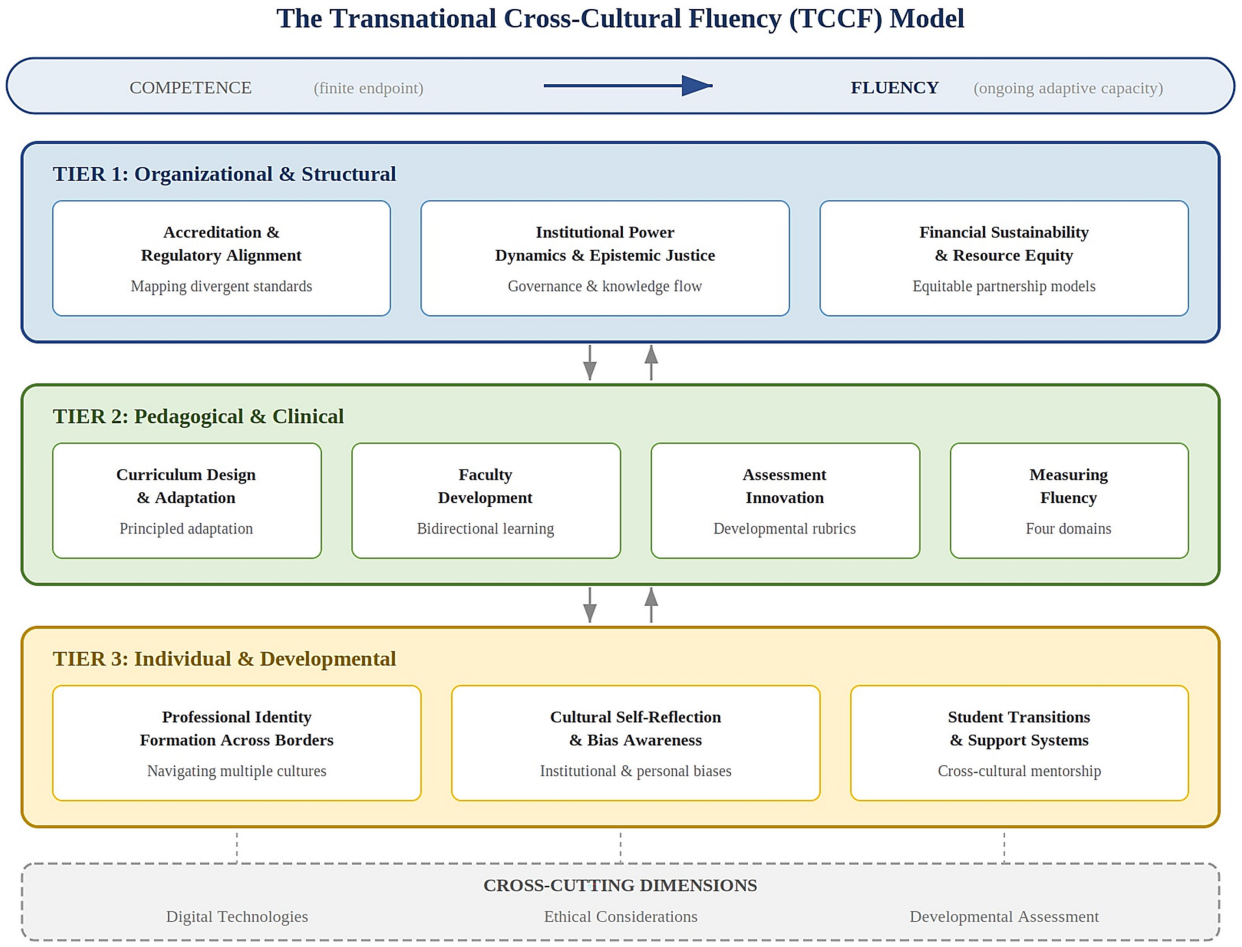
The transnational cross-cultural fluency (TCCF) model. The three-tier framework addresses organizational and structural barriers (Tier 1), pedagogical and clinical challenges across multiplied cultural contexts (Tier 2), and individual-level processes of identity formation and cultural adaptation (Tier 3). Cross-cutting dimensions—digital technologies, ethical considerations, and developmental assessment—span all three tiers. Bidirectional arrows indicate the interdependence of tiers, reflecting the model’s integrative architecture. The overarching trajectory from competence (finite endpoint) to fluency (ongoing adaptive capacity) is represented as the conceptual orientation governing the entire framework.

To clarify its epistemological identity: the TCCF model is best understood as an integrative conceptual framework with implications for curricular design, assessment development, and governance reform. It draws on multiple theoretical traditions—cultural humility, structural analysis, cultural safety, transcultural care, developmental psychology, and partnership ethics—but organizes them within a three-tier architecture specifically designed for the transnational medical education context. The model is not itself a curricular template or an assessment instrument, though it specifies the domains within which curriculum, assessment, and governance strategies should be developed. Its primary contribution is conceptual: providing a structured analytical lens through which the multi-layered cultural transactions of transnational medical education can be understood, addressed, and evaluated (Figure 1).

## The transnational cross-cultural fluency model

The TCCF model comprises three interdependent tiers, each addressing a distinct level of analysis in transnational medical education: (1) organizational and structural, (2) pedagogical and clinical, and (3) individual and developmental. This architecture builds on and extends Betancourt’s (6) tripartite framework for cross-cultural medical education by adapting it to the specific demands of transnational contexts.

## Tier 1: organizational and structural barriers in cross-border partnerships

Transnational medical education programs operate at the intersection of multiple institutional, regulatory, and governance systems, each of which embeds its own cultural assumptions about the purposes and standards of medical training. Addressing the structural conditions under which cross-cultural fluency can develop is therefore a prerequisite for effective programming at any level (24, 58).

### Accreditation and regulatory alignment

A fundamental structural challenge is the divergence between accreditation standards across jurisdictions. The LCME, the WFME, and the General Medical Council (GMC), among others, each articulate standards that reflect distinct national traditions regarding what constitutes adequate medical education (19, 28, 59). When a program must satisfy multiple accreditation bodies simultaneously—as is common in branch campus and dual-degree arrangements—faculty and administrators must navigate not merely different bureaucratic requirements but different philosophies of medical education, different assumptions about the balance between didactic and clinical instruction, and different expectations regarding the role of cultural education in physician training (58, 60). We propose that transnational programs develop explicit “accreditation mapping” documents that identify areas of convergence and divergence between applicable standards and that establish transparent processes for resolving conflicts, ideally in partnership with the accreditation bodies themselves.

### Institutional power dynamics and epistemic justice

Cross-border partnerships rarely involve institutions of equal standing, resources, or geopolitical influence (33, 35). When a prestigious university in a HIC establishes a branch campus or partnership in an LMIC, there is an inherent risk that the partnership will function as a conduit for unidirectional knowledge transfer rather than as a genuinely reciprocal exchange (36, 61). This dynamic has consequences for cross-cultural fluency: if the curriculum, pedagogy, and assessment methods are imported wholesale from the HIC institution, students receive implicit instruction that one cultural approach to medicine is authoritative while others are subordinate. Addressing this requires governance structures that ensure meaningful decision-making authority for all partner institutions, explicit mechanisms for incorporating local health knowledge and priorities into the curriculum, and regular audits of knowledge flow directionality within the partnership (62, 63).

### Financial sustainability and resource equity

The economic dimensions of transnational medical education also carry cultural implications. Programs in which students from LMICs pay fees commensurate with HIC tuition create educational environments that may not represent the socioeconomic diversity of the populations graduates will serve, thereby limiting students’ exposure to diverse perspectives (64). The economic burden of developing culturally responsive curricula must be addressed through creative financing mechanisms, including grants, partnerships with cultural organizations, government funding, and revenue-sharing models that ensure both partnering institutions benefit equitably (65, 66).

## Tier 2: pedagogical and clinical challenges across multiplied cultural contexts

The second tier addresses the educational core of transnational medical education: how curricula are designed, adapted, and delivered when the cultural distance between faculty, students, and patients spans national and continental boundaries.

### Curriculum design and contextual adaptation

Effective cross-cultural medical education in transnational settings requires curricula that are neither standardized imports from the home institution nor fully localized products that abandon the home institution’s pedagogical strengths (30, 67). Instead, a process of principled adaptation is needed, in which core learning objectives related to cross-cultural fluency are established collaboratively by partner institutions, while specific content, cases, and clinical scenarios are developed to reflect local epidemiological patterns, cultural health practices, and healthcare infrastructure (68). Medical schools have employed various approaches to integrating cross-cultural content, including standalone cultural competence courses, integration of cultural content across medical disciplines, and simulation-based learning with culturally diverse patient cases (6, 20, 69). In transnational contexts, these approaches must be extended to address not only patient–provider cultural differences but also the cultural dynamics within educational teams and between partnering institutions.

### Faculty development for international teaching environments

Faculty members who teach in transnational programs face unique challenges. They may be teaching in a language that is not their first, to students whose educational expectations and communication norms differ from their own, using clinical examples drawn from healthcare systems that neither they nor their students have directly experienced (70, 71). Existing calls for faculty development in cross-cultural medical education have emphasized cultural awareness, communication skills, and understanding of psychosocial determinants of health (6, 20). These remain essential, but in transnational contexts they must be supplemented with training in navigating educational culture shock, understanding different pedagogical traditions, and developing the capacity to learn from students who may possess local cultural knowledge that the faculty member lacks (72). We propose a faculty development framework specifically designed for transnational educators that incorporates self-reflection exercises adapted for contexts where the educator is the cultural outsider, rather than assuming the educator occupies a position of cultural familiarity

### Assessment innovation for cross-border settings

Traditional assessment methods may not capture the nuances of cross-cultural fluency in transnational settings (73). Written examinations developed in one cultural context may embed culturally specific assumptions about clinical reasoning, communication styles, and patient management that disadvantage students from other backgrounds (74). Objective structured clinical examinations (OSCEs) with cross-cultural scenarios, standardized patient interactions, and reflective portfolios represent promising approaches, but they require careful adaptation for transnational use (69). Assessment methods must be validated across the cultural contexts in which they will be deployed, standardized patients must reflect the diversity of patient populations in all relevant practice settings, and reflective assessment tools must be designed to evaluate the process of cultural engagement rather than the accumulation of cultural facts.

## Measuring cross-cultural fluency: toward developmental assessment

If fluency is conceptualized as a developmental capacity rather than a threshold competence, assessment must be correspondingly developmental. We propose a multi-method assessment approach organized around four interrelated domains of cross-cultural fluency.

First, cultural perspective-taking: the capacity to recognize, articulate, and critically evaluate how cultural systems, institutional norms, and epistemic traditions shape clinical reasoning and educational practice. Assessment approaches include structured reflective portfolios evaluated using developmental rubrics that track progression from surface-level cultural awareness to deep structural analysis of how culture operates in clinical and educational settings.

Second, adaptive communication: the ability to modify communication strategies effectively across cultural contexts, including awareness of nonverbal norms, hierarchical expectations, and language-mediated power dynamics. Cross-culturally validated OSCE stations, incorporating standardized patients from multiple cultural backgrounds, can evaluate not only the quality of communication but the learner’s capacity for adaptive reasoning when cultural expectations conflict.

Third, structural navigation: the capacity to identify, analyze, and constructively engage with institutional power asymmetries, regulatory differences, and governance structures that shape transnational educational partnerships. This domain is best assessed through case-based analyses and reflective exercises that require learners to identify structural barriers and propose contextually appropriate responses.

Fourth, reflexive practice: ongoing critical examination of one’s own cultural positioning, institutional assumptions, and epistemic biases as they influence professional behavior. Drawing on the Dreyfus model of skill acquisition (48), each domain can be assessed along a developmental continuum from novice (recognition of cultural difference) through advanced beginner (situational awareness), competent (contextual analysis), proficient (pattern recognition across cultural systems), to expert (intuitive cross-cultural navigation). Longitudinal tracking metrics—including graduate practice location, patient population diversity, and patient-reported cultural responsiveness—provide outcome-level data that complements process-level assessment.

Critically, assessment of fluency should capture trajectory and growth rather than fixed-point attainment, consistent with the conceptual shift from competence to fluency. The full psychometric development and cross-cultural validation of these assessment approaches exceeds the scope of this conceptual paper; however, instrument development and multi-site validation represent essential priorities for future empirical research.

## Tier 3: individual-level processes of identity formation and cultural adaptation

The third tier addresses the individual experiences of learners and educators who navigate multiple institutional and cultural contexts during their educational trajectory.

### Professional identity formation across borders

Medical students develop their professional identities through socialization into the norms, values, and practices of their educational institutions and clinical environments (75, 76). In transnational programs, this process occurs across multiple institutional cultures, potentially creating cognitive dissonance when norms conflict (77). A student completing preclinical education at a branch campus in one country and clinical rotations in another must integrate potentially divergent messages about the physician’s role, appropriate patient relationships, and the boundaries of professional authority. Programs should incorporate structured reflection opportunities and mentorship that explicitly address the challenge of forming a coherent professional identity across cultural contexts, framing this challenge as an opportunity for developing the adaptive capacity that characterizes cross-cultural fluency (78, 79).

### Cultural self-reflection and bias awareness in transnational contexts

The importance of self-reflection in cultural education is well established (6, 20, 80). Training that encourages providers to examine their own cultural identities, biases, and tendencies toward stereotyping can significantly enhance empathy and effectiveness in practice. In transnational settings, this work gains additional layers. Faculty and students must not only examine their personal cultural biases but also interrogate the institutional and epistemological assumptions they bring from their home educational cultures (81). Exercises and techniques that promote self-reflection—including open conversations exploring the impacts of racism, classism, sexism, and discrimination in healthcare; identifying hidden biases using patient vignettes; and discussing family interactions with healthcare systems (6, 20)—should be expanded to include reflection on how institutional cultures shape clinical reasoning, how accreditation standards embed cultural values, and how power dynamics within educational partnerships influence whose knowledge counts.

### Student transitions and support systems

Students in transnational programs frequently transition between institutional settings in different countries, a process that creates both pedagogical and personal challenges (82, 83). These transitions require support systems that go beyond orientation programs to include ongoing cross-cultural mentorship, peer support networks that span institutional boundaries, and academic advising that accounts for the specific challenges of navigating multiple educational cultures (84). Digital platforms can facilitate continuity of support across transitions, connecting students with mentors and peers regardless of geographic location, though care must be taken to ensure that digital mediation does not flatten or homogenize the cultural learning that transitions make possible (85).

## Illustrative application: the TCCF model in practice

To demonstrate the applied relevance of the TCCF model, consider a composite scenario drawn from patterns documented in the transnational medical education literature: a UK medical school establishing a dual-degree partnership with a university in sub-Saharan Africa.

At Tier 1 (organizational and structural), the TCCF framework would direct attention to accreditation mapping between the General Medical Council (GMC) and the relevant African regulatory body, identifying areas where standards converge and diverge—for example, differing expectations regarding the balance between didactic and clinical instruction, or differing requirements for community health exposure. Governance structures would be designed to ensure genuine shared decision-making authority—not merely consultative roles for the African institution—with explicit mechanisms for incorporating local health priorities into the jointly developed curriculum. Financial models would be scrutinized to ensure that tuition structures do not create educational environments unrepresentative of the populations graduates will serve, and revenue-sharing arrangements would be established to ensure equitable institutional benefit.

At Tier 2 (pedagogical and clinical), the framework would guide the collaborative development of a curriculum that integrates the UK institution’s pedagogical approaches (e.g., problem-based learning) with local epidemiological realities, cultural health practices, and healthcare infrastructure. Crucially, this is not a one-way adaptation: problem-based learning itself, developed within Western pedagogical traditions, carries cultural assumptions about self-directed learning, peer critique, and student–faculty relationships that may not transfer seamlessly to educational cultures with different hierarchical norms and collaborative traditions (30). Faculty development would be bidirectional: UK faculty would receive structured preparation in navigating educational culture shock and learning from local clinical knowledge, while African faculty would engage with the UK institution’s pedagogical approaches on terms of epistemic equality rather than hierarchical transfer. Assessment methods—including OSCE scenarios and reflective exercises—would be co-developed and validated across both cultural contexts, with multi-cultural rater panels ensuring that cultural bias does not systematically disadvantage students from either setting.

At Tier 3 (individual and developmental), the program would implement structured reflection opportunities for students who must integrate potentially divergent professional identity messages across their two institutional contexts. Cross-cultural mentorship pairings—connecting students with mentors from both partner institutions—would support identity formation. Assessment of cross-cultural fluency across the four proposed domains (cultural perspective-taking, adaptive communication, structural navigation, and reflexive practice) would track students’ developmental trajectories longitudinally, providing formative feedback to learners and outcome data for ongoing program refinement.

This illustrative application demonstrates how the TCCF model’s three-tier architecture provides a structured approach to the complex, interdependent decisions that transnational partnerships face, moving beyond general principles of cultural sensitivity toward specific, actionable, and level-appropriate interventions.

## Digital platforms and technology-enhanced education in transnational CCME

Digital technologies play an increasingly central role in transnational medical education, serving as both delivery mechanisms for cross-border curricula and tools for developing cross-cultural fluency (86, 87). Technology-driven simulations can expose students to diverse clinical scenarios across cultural contexts, while real-time feedback systems using culturally diverse standardized patients can provide assessment data that transcends geographic limitations (69, 88).

Recent work has advanced the definition of digital health competencies for global health contexts. Car et al. (89) provide a comprehensive framework for digital health education that addresses the knowledge, skills, and attitudes required for effective technology-mediated healthcare delivery across diverse settings. Their contribution demonstrates the growing scholarly attention to ensuring that digital health education is fit for purpose in globalized healthcare. The TCCF model extends this work by situating digital health competencies within the broader cultural dynamics of transnational education: when digital health education is delivered across borders, the cultural assumptions embedded in technology platforms, assessment algorithms, and communication norms must be negotiated across multiple cultural systems simultaneously—a challenge that requires not only technical digital competence but the cross-cultural fluency to recognize and navigate the cultural dimensions of digital health tools themselves.

However, digital platforms also introduce their own cultural complexities. Learning management systems, virtual patient simulations, and telehealth training tools are typically developed within specific cultural contexts and may embed assumptions about learning styles, communication norms, and clinical interaction that do not translate seamlessly across cultures (90, 91). The design of digital educational tools for transnational use requires input from all partner cultures to ensure cultural validity, and the use of artificial intelligence in educational assessment must be scrutinized for cultural bias (92, 93).

Telehealth and remote clinical education have emerged as particularly relevant for transnational programs, enabling students to participate in clinical encounters across cultural contexts without physical relocation (94). These modalities offer new opportunities for cross-cultural clinical exposure but also present challenges, including the difficulty of learning culturally appropriate nonverbal communication through screens and the risk that remote engagement provides only a superficial cultural experience that lacks the immersive quality necessary for developing genuine fluency (95, 96).

## Ethical considerations and the imperative of health equity

The ethical dimensions of transnational medical education extend beyond the standard considerations of informed consent and institutional review (97). Programs that operate across borders must grapple with questions of epistemic justice: whose medical knowledge is valued, whose cultural health practices are recognized as legitimate, and whose perspectives shape the curriculum (35, 61, 98). The risk of cultural hegemony—in which the educational practices of dominant institutions displace local traditions without adequate justification—is a persistent concern that must be addressed through deliberate structural safeguards rather than left to individual goodwill (36, 99).

As Abimbola (100) has argued, authorial positionality shapes the framing of global health scholarship, and single-perspective work risks reproducing the very asymmetries it seeks to critique. The present framework was developed from a Global North institutional position, and while the author’s own cross-cultural trajectory—spanning multiple European and North American institutions with direct experience of the cultural negotiations inherent in transnational education—provides experiential grounding, this does not substitute for the systematic inclusion of diverse scholarly and practitioner voices. Future refinement and empirical validation of the TCCF model should be undertaken through collaborative partnerships with educators and institutions situated across the full range of global contexts in which transnational medical education operates.

Health equity is both a driving motivation and an ethical standard for transnational medical education. Programs should be evaluated not only by the quality of education they deliver but also by their contribution to equitable health outcomes in all communities they touch (37, 101). This evaluation requires longitudinal tracking of graduates’ practice patterns, including where they practice, whom they serve, and how effectively they navigate cultural diversity in clinical encounters. If transnational programs systematically produce graduates who migrate to HICs rather than serving LMIC populations, the programs may be exacerbating rather than alleviating global health inequities, regardless of the quality of their cross-cultural curriculum (102, 103).

Failure to address sociocultural factors in medical education, whether in domestic or transnational settings, can lead to stereotyping, biased or discriminatory treatment of patients, and the perpetuation of health disparities (6, 8). Transnational programs bear a heightened responsibility to ensure that their educational models do not merely reproduce existing inequities at a global scale but actively contribute to a more equitable distribution of culturally fluent healthcare providers.

## Limitations

Several limitations of this work warrant acknowledgment. First, the TCCF model is a theoretical framework that has not yet been empirically tested or validated in practice settings. While grounded in established literature across cross-cultural medical education, transnational education governance, and global health workforce research, its practical utility, feasibility, and effectiveness remain to be demonstrated through implementation research across diverse transnational partnerships. Until such evidence is generated, the model should be understood as a conceptual contribution that requires empirical refinement.

Second, the framework was developed from the perspective of a single author based at a high-income country institution. As Abimbola (100) has argued, authorial positionality shapes the framing of global health scholarship, and single-perspective work risks reproducing the asymmetries it seeks to critique. While the author’s own transnational academic trajectory—spanning multiple European and North American institutions with direct experience of cross-cultural educational negotiations—provides experiential grounding, this does not substitute for systematic collaborative development with scholars and practitioners situated across the full range of global contexts.

Third, the reference base, while efforts have been made to strengthen engagement with LMIC-authored scholarship, still reflects the broader publication asymmetry in medical education scholarship that favors HIC-based authors and English-language outlets. More inclusive citational practice and deeper engagement with non-Anglophone scholarship represent important commitments for future iterations of this work.

Fourth, the assessment approaches proposed for cross-cultural fluency—including developmental rubrics, reflective portfolios, cross-culturally validated OSCEs, and longitudinal tracking metrics—remain conceptual. Their psychometric development, cross-cultural validation, and practical feasibility require dedicated empirical research programs.

Fifth, the model does not address all forms of transnational medical education equally. It is most directly applicable to formal institutional partnerships, branch campus arrangements, and dual-degree programs. Less formalized transnational educational encounters—including short-term clinical rotations, visiting faculty exchanges, and digital learning collaborations—may require adapted applications of the framework’s principles. Practical implementation guidance and tiered recommendations based on partnership maturity and resource availability would strengthen the model’s accessibility and should be developed through future collaborative work.

Sixth, although this paper has clarified the framework’s applicability across health professions, the current analysis is grounded primarily in medical education. Adaptation and validation for nursing, allied health, and interprofessional education contexts remain important future directions.

## Conclusion

The globalization of medical education has created both opportunities and imperatives for reimagining how healthcare professionals are prepared to deliver culturally responsive care. The Transnational Cross-Cultural Fluency model proposed in this paper provides a structured framework for addressing the multi-layered cultural, institutional, and ethical challenges that characterize medical education across borders. By organizing analysis and intervention across three tiers—organizational, pedagogical, and individual—the TCCF model offers a comprehensive approach that complements and extends existing cultural competence frameworks.

The conceptual shift from competence to fluency is not merely semantic. It signals a fundamental reorientation from the acquisition of discrete cultural knowledge toward the development of ongoing adaptive capacity. In a world where healthcare professionals may train in one country, practice in another, and serve patient populations drawn from dozens of cultural backgrounds, the ability to engage respectfully and effectively with cultural unfamiliarity is more valuable than expertise in any particular cultural tradition. Cross-Cultural Medical Education has become an imperative, and its transnational extension is now of paramount importance to satisfy the need for effective cross-cultural communication and understanding in patient care across a globalized healthcare landscape.

Implementation of the TCCF model will require coordinated effort across multiple stakeholders. Partnering institutions must develop governance structures that ensure bidirectional knowledge exchange and genuine shared decision-making. Accreditation bodies must evolve their standards to account for the realities of cross-border education, potentially developing transnational accreditation pathways that respect cultural variation while maintaining educational quality. Faculty development programs must be reconceived for educators who teach across cultural contexts, equipping them not merely with cultural knowledge but with the humility and adaptive capacity to learn from the cultural contexts in which they teach. Assessment methods must be reimagined to evaluate cross-cultural fluency as a developmental process rather than a fixed outcome.

Critically, this refinement process must itself embody the principles of epistemic justice and bidirectional exchange that the TCCF model advocates. Participatory, multi-site development involving educators, learners, and administrators from LMIC institutions—not as consultants but as co-architects—is essential to ensure that the framework reflects the full range of perspectives and contexts it seeks to serve. The TCCF model’s applicability across health professions education further underscores the need for cross-disciplinary collaboration in its future development.

Medical educators worldwide must remain committed to incorporating feedback, evolving best practices, and innovative teaching strategies to ensure that future healthcare professionals are well-equipped to provide high-quality, culturally fluent care to all patients. The stakes are considerable: in a globally interconnected healthcare landscape, the quality of cross-cultural medical education shapes not only individual clinical encounters but the broader pursuit of health equity across communities, nations, and continents.

## Data Availability

The original contributions presented in the study are included in the article/supplementary material, further inquiries can be directed to the corresponding author/s.
